# Does Valved Holding Chamber Improve Aerosol Lung Deposition with a Jet Nebulizer? A Randomized Crossover Study

**DOI:** 10.3390/pharmaceutics14030566

**Published:** 2022-03-04

**Authors:** Luciana Alcoforado, Dulciane Nunes Paiva, Arzu Ari, Jacqueline de Melo Barcelar, Simone Cristina Soares Brandão, James B. Fink, Armele Dornelas de Andrade

**Affiliations:** 1 Department of Physical Therapy, Universidade Federal de Pernambuco, Recife 50710-560, PE, Brazil; alcoforadoluciana@gmail.com (L.A.); jacqueline_barcelar@hotmail.com (J.d.M.B.); 2 Department of Physical Education and Health, Universidade de Santa Cruz do Sul, Santa Cruz do Sul 96815-010, RS, Brazil; dulciane@unisc.br; 3 Department of Respiratory Therapy, Texas State University, Austin, TX 73301, USA; arzuari@hotmail.com; 4 Medicine Nuclear Department, Universidade Federal de Pernambuco, Recife 50710-560, PE, Brazil; sbrandaonuclearufpe@gmail.com; 5 Department of Cardiopulmonary Sciences, Division of Respiratory Care, Rush University Medical Center, Chicago, IL 60007, USA; fink.jim@gmail.com

**Keywords:** vibrating mesh nebulizer, jet nebulizer, scintigraphy, spacer, valved holding chamber, aerosol delivery

## Abstract

Using valved holding chambers (VHC) during aerosol therapy has been reported to improve the inhaled dose with various aerosol devices, including vibrating mesh nebulizers. The aim of this study was to quantify the pulmonary deposition of a jet nebulizer (JN) with and without a VHC, and a mesh nebulizer (MN) with a VHC in a randomized cross-over trial with seven healthy consenting adults. Our hypothesis was that the use of a VHC would improve deposition with the JN. Diethylnitriaminopentacetic acid with technetium (DTPA-Tc99m), with the activity of 1 mC with 0.9% saline solution was nebulized. The radiolabeled aerosol was detected by 2D planar scintigraphy after administration. The pulmonary deposition was greater with a JN with a VHC (4.5%) than a JN alone (3.2%; *p* = 0.005. However, an MN with a VHC (30.0%) was six-fold greater than a JN or JN with a VHC (*p* < 0.001). The extrapulmonary deposition was higher in the JN group without a VHC than in the other two modalities (*p* < 0.001). Deposition in the device was greater with a JN + VHC than an MN+/VHC (*p* < 0.001). Lower residual drug at the end of the dose was detected with an MN than either JN configuration. The exhaled dose was greater with a JN alone than either an MN or JN with VHC (*p* < 0.001). In conclusion, the addition of the VHC did not substantially improve the efficiency of aerosol lung deposition over a JN alone.

## 1. Introduction

Valved holding chambers (VHC) have been reported to improve the inhaled dose of medical aerosols with a variety of aerosol devices, including vibrating mesh nebulizers (MN) [1,2,3]. VHCs were designed to provide a reservoir to collect aerosol between inhalations to improve the pulmonary deposition of inhaled medications in spontaneously breathing patients.

In vivo and in vitro studies report greater aerosol delivery using vibrating mesh nebulizers when compared to jet nebulizers (JN) [4,5,6,7,8]. These advantages are related to the physical characteristics of this device, such as low residual volume, no gas flow required to generate the aerosol and minimal disruption of ventilation compared with JNs [3,8,9,10].

Developed initially for use with pressurized metered-dose inhalers (pMDIs), the VHC has been well established to improve inhaled doses while reducing dependency on inhaler techniques with performance based on the VHC design [11].

The potential benefits of VHC, when used in conjunction with nebulizers, has not been well established. The combination of MN with VHC is in clinical use with in vitro and in vivo reports of higher inhaled doses than standard JNs [1,2]. Despite the advantages of an MN, JNs are widely used in the emergency department and acute care settings, due in part to their long-standing use and their low cost [12].

The performance of JNs has been shown to improve with the use of a tubing reservoir [13], raising the question of whether delivery efficiency could be further improved by the use of a VHC [1,2]. Few studies in the literature assessed the deposition of aerosols when using these adapters/interfaces with a JN. Sarhan et al. reported a significantly higher amount of delivered aerosol using a VHC with a JN and MN than the same nebulizer with a T-piece [2]. Thus, the authors recommended using the VHC with both the JN and MN for better aerosol delivery.

Although the use of MNs with a VHC device has been reported to deliver greater inhaled doses than JNs [1,14], no studies have compared pulmonary deposition by scintigraphy of radiolabeled aerosol particles generated by a JN in a constant output with and without a VHC in healthy subjects. We hypothesize that the use of a JN with a VHC would improve lung deposition over a JN alone.

## 2. Materials and Methods

### 2.1. Study Population

We recruited healthy volunteers between 18 and 60 years of age, with a forced vital capacity (FVC), and a forced expiratory volume; on the first (FEV_1_) ≥80% of predicted and who have the ability to understand verbal commands [14]. Individuals with a history of pulmonary diseases, pregnant women, the elderly, and smokers were excluded.

**Figure 1:**
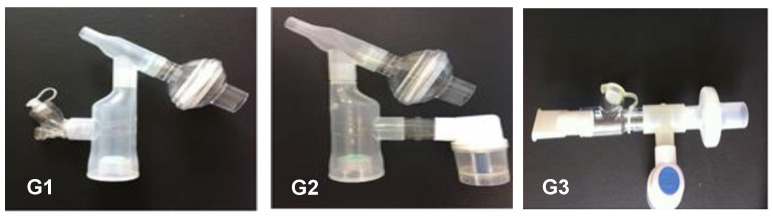
Nebulizer configurations used in this study. G1: mesh nebulizer with VHC, G2: jet nebulizer with VHC, and G3: jet nebulizer alone.

Consented subjects were randomized into three groups according to the type of nebulization procedures: (G1) MN with VHC (MN + VHC); (G2) JN with VHC (JN + VHC) and (G3) JN alone (JN without VHC) (JN) (Figure 1). The order of the three procedures for each subject was randomized using a table generated on the site randomization.com and placed in opaque envelopes to ensure blinding. All researchers were trained to perform the tests and use the instruments as per protocol, in accordance with the quality criteria. Data analyses were performed by staff who were blinded to administration arms to subjects.

### 2.2. Study Design

This cross-over randomized clinical trial was performed at the Laboratory of Cardiopulmonary Physiotherapy and the Department of Nuclear Medicine of Hospital das Clínicas de Pernambuco of the Federal University of Pernambuco, in Recife, Pernambuco, Brazil. The study was approved by the Research Ethics Committee of the UFPE (CAAE: 44794415.4.0000.5208; Clinical Trials: NCT: 02501655), and all participants signed a written informed consent.

The sample size was calculated using software developed by the Mallinckrodt General Clinical Research Center, based on the results of the first five volunteers, for a statistical power of detecting differences between procedures of 80% and a significance level of 0.05. A sample size of six volunteers, minimum, was determined to be appropriate for this study.

### 2.3. Measurements

Anthropometric characteristics and vital signs (heart rate (HR), respiratory rate (RR), peripheral oxygen saturation (SpO_2_%), and blood pressure (BP)) were collected. A digital and portable spirometer (MicroLoop^®^, Cardinal Health, Kent, UK) was used to evaluate lung function according to American Thoracic Society criteria [15]. An MN (Aerogen Solo: Aerogen Ltd., Galway, Ireland) and a JN (NS Medical Device Industry Ltda., São Paulo, Brazil) were used to administer aerosol, either directly or through a VHC (Ultra: Aerogen Ltd., Galway, Ireland). The volunteers performed the nebulization procedures with an MN + VHC (G1), JN + VHC (G2), and a JN alone (G3), as shown in Figure 1. Aerosol administration was separated by a washout period of 24 to 42 h.

The inhalation of the radioaerosol was performed according to the methods described by Galindo-Filho et al. using diethylnitriaminopentacetic acid with technetium (DTPA-Tc99m), with an activity of 1 mC with 0.9% saline solution to a total volume of 2 mL (guideline according to the manufacturer) for an MN and 4 mL for a JN. The oxygen driving flow that was used to operate a JN was 8 L/min [8].

Prior to each dose, the subject was introduced to the device, instructed to seal their lips around the mouthpiece and breathe quietly during the course of administration. The dose was placed into the nebulizer, and aerosol was administered. After each aerosol administration was complete, the volunteer was escorted to the room for scintigraphic images acquisition and was positioned in front of a collimator (Starcam 3200 GE, Little Chalfont Buckinghamshire, UK) at a distance of 30 cm from the midline of the humeral head to the collimator. Images of the posterior thorax, face and circuit were acquired with a period of 300 s for each image obtained, with a matrix of 256 × 256. The total duration of scintigraphic acquisition took 30 min.

The Xeleris 3 Functional Imaging Workstation (GE Healthcare, Milwaukee, WI, USA) software was used to analyze the images with four regions of interest (ROI): (1) lungs, (2) extrapulmonary (i.e., upper airway, and stomach), (3) device (i.e., nebulizer, VHC or T-piece), and (4) expiratory filter. The sum of counts from each compartment was combined to determine the mass balance.

The lung deposition of the upper respiratory tract, stomach and circuit components was combined in a cumulative count representing the total mass of radioaerosol. Each compartment was expressed as a percentage of the total [16].

### 2.4. Statistical Analysis

Data were analyzed using the Statistical Package for the Social Sciences, version 24.0 (IBM Inc., New York, NY, USA). Descriptive statistics, including the mean and standard deviations, were calculated. The Shapiro–Wilks test was used to analyze the normality of the data and the Levene test to verify the homogeneity. Pulmonary and extrapulmonary aerosol depositions were expressed as a percentage of the nominal and emitted dose, respectively. The emitted dose was equal to the nominal dose minus the dose recovered in the reservoir after the inhalation. The aerosol output rate was calculated by the ratio of lung dose to the duration of nebulization. The Kruskal–Wallis test was used to evaluate the pulmonary deposition and ROI between the nebulization procedures and the Tukey post hoc test to identify the differences between groups (*p* < 0.05).

The primary outcome of our study was to quantify and compare pulmonary deposition between a JN alone, the JN/VHC, and the MN/VHC. The secondary outcome was the assessment of extrapulmonary (upper airways, stomach) and device deposition (nebulizer, T-piece adapter, chamber and expiratory filter) deposition.

## 3. Results

**Figure 2:**
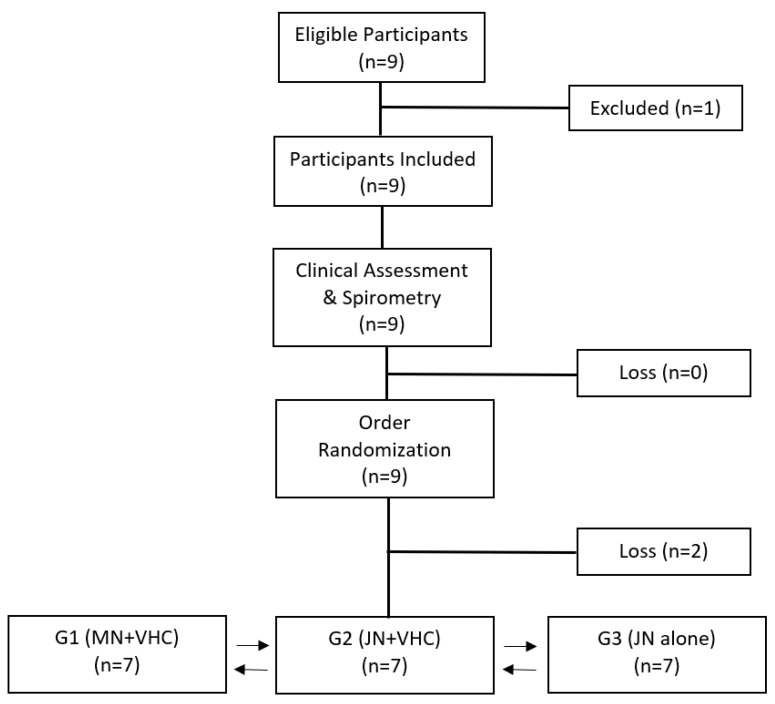
Volunteer recruitment and follow-up flowchart according to the CONSORT (Consolidated Standards of Reporting Trials) statement. MN + VHC: mesh nebulizer with the valved holding chamber, JN + VHC: jet nebulizer with the valved holding chamber, and JN alone: jet nebulizer without the VHC.

Ten volunteers were recruited and consented, with three dropouts (one subject was pregnant, while one subject was uncomfortable with nebulization, and the other did not complete the study). Seven volunteers (*n* = 7), including 4 women and 3 men, completed the study (Figure 2). The anthropometric and spirometric characteristics of the individuals are shown in Table 1.

**Figure 3:**
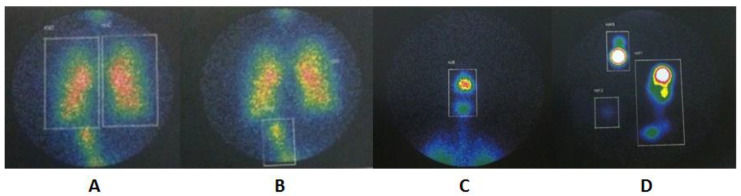
Pulmonary and extrapulmonary ROIs that are used to quantify aerosol deposition and calculated the mass balance across compartments including (**A**) total lung regions, (**B**) stomach, (**C**) upper airways, (**D**) nebulizer, adapter and expiratory filter.

**Figure 4:**
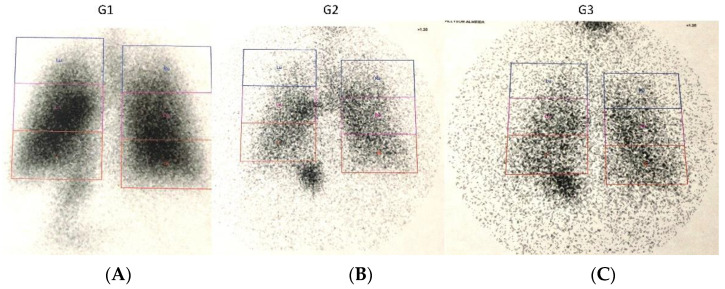
Representative images of posterior thorax post-nebulization with each device configuration. (**A**) G1: Mesh nebulizer with VHC, (**B**) G2: Jet nebulizer with VHC, (**C**) G3: Jet nebulizer alone (Jet nebulizer without VHC).

**Figure 5:**
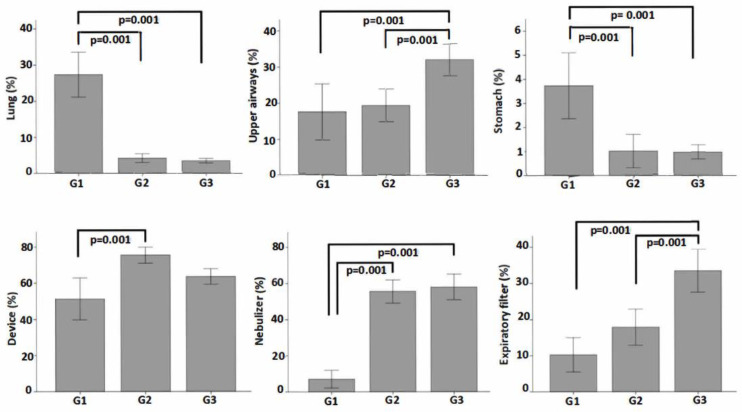
Graphical analysis of the difference between each device configuration with a mean (±SD) % deposition in the lungs, upper airways and stomach, device (including nebulizer, VHC or T-piece), nebulizer alone, and expiratory filter. G1: mesh nebulizer with VHC, G2: jet nebulizer with VHC, G3: jet nebulizer alone.

An illustration of pulmonary, extrapulmonary, and device ROI is shown in Figure 3. The results of the proportion of total aerosol represented in each ROI are presented in Table 2 and represented in Figure 4. A graphical analysis of the difference between the three device configurations in aerosol deposition to the lungs, upper airways, stomach, device total, nebulizer alone, and expiratory filter is shown in Figure 5.

The pulmonary deposition was greater with an MN with a VHC than the JN with and without a VHC (*p* < 0.001). The extrapulmonary aerosol deposition was higher with a JN without a VHC when compared to the other two modalities (*p* < 0.001), while we found no statistical or clinically relevant difference between the MN + VHC and the JN + VHC (Figure 5). Stomach deposition was greater with an MN + VHC compared to the JN with (*p* < 0.001) and without a VHC (*p* = 0.001) (Figure 4 and Figure 5).

Total device deposition was greater for a JN + VHC and a JN alone than the MN + VHC (*p* < 0.001). The residual volume remaining in the nebulizer at the end of administration was lower in an MN + VHC than either JN iteration. Radiation in the expiratory filter was greater with the JN group without a VHC than either the JN or MN with a VHC (*p* < 0.001).

## 4. Discussion

To the best of our knowledge, this is the first study to demonstrate the impact of administration of radiolabelled aerosol from a VHC with a JN. While the use of a VHC increased lung dose by 40% over the JN alone, the change from 3.2% to 4.5% is not likely to be of clinical consequence. In contrast, the pulmonary deposition with an MN + VHC was six-fold more efficient than either JN configuration.

Our findings are consistent with Dugernier et al. [1] using SPECT-CT analysis and reported a six-fold greater pulmonary aerosol deposition with an MN compared to the JN with tubing (34.1 ± 6.0% vs. 5.2 ± 1.1%, *p* < 0.001). Differences in absolute values of deposition are small. They may be partly due to differences in the design of JNs and the methods of analysis used in this study.

Similarly, in an in vitro study Ari and colleagues [14] reported a similar inhaled dose of 7.66% with a JN attached to a tubing reservoir and 34.99% with an MN + VHC measured distal to the bronchi of an airway model. The inhaled dose with an MN + VHC was 4.5 times greater than the JN. Sarhan et al. [2] reported in vitro findings that the delivery efficiency of an MN + VHC (43%) was greater than the JN (9%). However, when a JN was used with the VHC, the emitted dose doubled to 19.1%. This is in sharp contrast with our in vivo findings of lower pulmonary deposition and only a 40% increase with the JN + VHC.

The VHC used in this study consists of a 150 mL tube with an inlet port for the nebulizer near the center. It has an inspiratory valve at the base of the chamber and an expiratory valve positioned immediately across from the mouthpiece (Figure 1). With the VMN, aerosol enters the chamber continuously. During inhalation, the inspiratory valve of the VHC opens and the expiratory valve closes to allow aerosol to be cleared from the chamber. During exhalation, the inspiratory valve is closed while the patient exhales through the mouthpiece, allowing aerosol that has entered the chamber to remain undisturbed between breaths. The VHC acts as a reservoir where aerosol collects between inspirations. While in the chamber, aerosol can rain out largely due to sedimentation, as evidenced by the greater loss of aerosol in the VHC with G1 (device minus nebulizer).

In contrast, the JN is driven by an operating flow of 8 L/min (133 mL/s). Emitted aerosol is inhaled during inspiration and then continues to flow into the chamber between breaths. However, the continuous gas flow fills and overflows the small chamber and passes through the expiratory valve to the atmosphere, reducing the aerosol available for inspiration, as evidenced by the 19% and 32.25% of the dose collected in the expiratory filter for a JN + VHC and JN alone, respectively. The effect of gas flow in the VHC is not unique to the JN. When 8 L/min of oxygen is administered through the VHC, the inhaled dose decreases by more than 50% [14,17]. This suggests that optimal pulmonary deposition can be achieved with inspiration through an MN + VHC with low flow oxygen (<10 L/min) administered by a low flow nasal cannula. A typical peak inspiratory flow >20 L/min allows clearance of the 150 mL chamber with each breath.

Our findings are in agreement with those of Dugernier et al. [1] who observed a greater aerosol collection in the expiratory filter when using the JN. In our study, the highest percentage of deposition in the expiratory filter was observed in the JN group without a VHC compared to the other groups, indicating a greater loss of aerosol between inhalation cycles.

Regarding deposition in the upper airway and stomach, our findings are consistent with reports of Dugerneir et al. [1] and Galindo et al. [8]. In both studies, a higher percentage of radioaerosol was found in the stomach and airways when using MNs compared to JNs. Dugernier et al. [1] observed 14.5% and 4.6% deposition in the stomach and upper airway respectively when performing nebulization with MNs, compared to 1.6% and 0.6% with the JN. In contrast, Galindo et al. [8] observed 1.4% and 16.2% in the stomach and upper airway with the MN vs. 16.2% and 4.3% with the JN. The increase in aerosol deposition may be related to the greater overall inhaled dose with the MN [1,8].

In addition to pulmonary and extrapulmonary deposition, it is important to evaluate radiation deposited in all compartments. For instance, aerosol deposition in the device comprises a nebulizer (residual volume), VHC or T-piece with a mouthpiece and expiratory filter that corresponds to the actual technical effects of the nebulizer with or without a VHC to evaluate losses of aerosol that are not delivered to the patient. Our results showed a smaller amount of radiolabeled drug retained in a nebulizer (residual volume) with less exhaled aerosol in the filter when an MN+VHC was used, with the greatest loss of aerosol in a VHC. Our findings of low residual volume with the MN agree with other studies. Galindo et al. [8] reported only 5.08% of the solution volume remained in an MN compared to 41.29% in the JN. Mc Peck et al. [18] reported residual volume values of 3.49% in an MN and 54.9% in a JN, while Tiemersma et al. [19] reported that the MN had a smaller volume of solution retained in a nebulizer (13.9%) compared to the JN (64.9%). Dugernier et al. [1] found 2.4% residual volume with the MN and 62.8% for the JN. Sarhan et al. [2] reported a residual volume of 3467.1 μg (69% of a 5000 μg 1 mL dose) with the JN + VHC versus 186.8 μg (3.7%) with an MN. In this case, the higher residual volume in the JN alone and the JN + VHC might be attributed to the low dose volume of 1.0 mL used. With a 3 mL dose in the same nebulizer, a greater proportion of the dose would be emitted, presumably with a similar residual of drug remaining in the nebulizer at the end of the dose.

Some limitations have to be considered. We did not correct the tissue absorption of radiation, which means we may have underestimated pulmonary and extrapulmonary deposition and overestimated the device measurements. Nonetheless, our findings were consistent with previous in vitro reports. We only evaluated one type of VHC with an internal volume of 150 mL, previously characterized in both in vitro and in vivo studies. Although VHCs with different internal volumes may have a different effect on aerosol delivery, the premise of continuous gas flow overflowing the VHC should limit the effectiveness with JNs. This study was performed on healthy subjects. Therefore, their lung deposition cannot be extrapolated directly to subjects with pulmonary diseases, such as asthma, *chronic obstructive pulmonary disease*, bronchiectasis, and cystic fibrosis, in whom obstructive airways tend to retain a higher percentage of inhaled aerosol, as previously observed with both the JN and MN.

## 5. Conclusions

The addition of a VHC to JNs did not result in clinically significant improvement in pulmonary drug delivery. Our results demonstrate six-fold higher radioaerosol deposition in the pulmonary region using MNs with a VHC compared to JNs with or without a VHC.

## Figures and Tables

**Table 1:** Means and standard deviations (SD) of anthropometric and clinical characteristics of study participants. (BMI: body mass index; HR: heart rate; BPM: beats per minute; RR: respiratory rate; SpO_2_: peripheral oxygen saturation; FEV_1_: forced vital capacity; PEF: peak expiratory flow; and FEF_25–75%_: forced expiratory flow between 25–75%.).

Variable	Mean	SD
Gender (Male/Female)	¾	
Age (year)	24	6.69
BMI (kg/m^2^)	25.77	3.62
HR (bpm)	83	6.37
RR (breath per minute)	14.67	3.98
SpO_2_ (%)	97.80	1.09
FEV_1_ (% pred)	84.83	8.63
FVC (% pred)	81.67	11.39
FEV_1_/FVC	102.50	9.33
PEF (% pred)	80.75	5.31
FEF_25–75%_ (% pred)	85.5	13.66

**Table 2:** Median and interquartile range (IQR) of aerosol deposition measured in the lungs, upper airways, stomach, the device (including nebulizer, VHC or T-piece), nebulizer alone, and expiratory filter. (G1: mesh nebulizer with VHC, G2: Jet nebulizer with VHC, G3: Jet nebulizer alone).

	G1 (*n* = 7) Median (IQR)	G2 (*n* = 7) Median (IQR)	G3 (*n* = 7) Median (IQR)	*p*-Value
Lungs (%)	30.02 (16.2–35.3)	4.5 (2.7–5.0)	3.24 (2.7–4.5)	0.001
Upper airways (%)	17.35 (13.2–20.6)	20.82 (13.4–24.4)	33.9 (25.2–39.4)	0.001
Stomach (%)	3.52 (3.0–7.1)	0.82 (0.5–1.3)	1.34 (0.5–1.5)	0.002
Device (%)	53.27 (39.4–60.93)	75.7 (70.4–80.5)	62.75 (55.2–70.4)	0.001
Nebulizer (%)	4.83 (2.3–11.2)	56.99 (51.2–60.3)	50.23 (46.3–52.6)	0.001
Expiratory filter (%)	13.17 (9.6–5.4)	19.17 (12.1–23.5)	32.25 (23.6–38.1)	0.001

## Data Availability

The data and contributions presented in the study are included in the article. Further inquiries can be directed to the corresponding author.
